# Transthoracic Echocardiography of the Neonatal Laboratory Piglet

**DOI:** 10.3389/fped.2019.00318

**Published:** 2019-07-31

**Authors:** Stephan Schwarz, Miriam Kalbitz, Helmut D. Hummler, Marc R. Mendler

**Affiliations:** ^1^Division of Neonatology and Pediatric Critical Care, Department of Pediatrics and Adolescent Medicine, Ulm University, Ulm, Germany; ^2^Department of Traumatology, Hand-, Plastic-, and Reconstructive Surgery, Center of Surgery, Ulm University, Ulm, Germany; ^3^Division of Neonatology, Department of Pediatrics, Sidra Medicine, Doha, Qatar

**Keywords:** neonatal transthoracic echocardiography, piglets, hemodynamic monitoring, ventricular function, swine

## Abstract

**Background:** Newborn piglets are commonly used in biomedical research. However, cardiovascular imaging of this species is quite challenging. For point of care diagnostics of heart function transthoracic echocardiography may be used, which appears to differ comparing newborn piglets with adult pigs. To date, there are few data or studies on the feasibility and quality of measurement of functional echocardiographic parameters in very small neonatal piglets.

**Objectives:** To study the feasibility of transthoracic echocardiography in very small newborn piglets in supine position.

**Methods:** In 44 anesthetized and intubated newborn piglets, positioned in supine position [age 32 h (12–44 h), weight 1,220 g (1,060–1,495 g), median (IQR)] transthoracic echocardiography was performed using a point of care ultrasound device (M-Turbo^©^, FujiFilm SonoSite BV, Amsterdam, Netherlands), and a standard ultrasound transducer.

**Results:** Using 2D- and M-mode-imaging left- and right-sided heart structures were accessible to transthoracic echocardiography in neonatal piglets. Diameters of the interventricular septum, the left ventricle, and the posterior wall were measured and ejection fraction and shortening fraction was calculated. Both left and right ventricular outflow tract could be imaged, and ventricular filling and systolic function could be evaluated. Furthermore, we were able to assess shunts of fetal circulation, such as patent ductus arteriosus, structure of the heart valves and congenital heart defects including ventricular septal defect.

**Conclusions:** In summary, transthoracic echocardiography is feasible for assessment of cardiovascular function even in very small newborn laboratory piglets in supine position.

## Introduction

Swine is one of the larger animal species commonly used in biomedical research. Many of their anatomical and physiological features are comparable to humans (1). Therefore, assessment of cardiac function is useful for many research questions. Invasive continuous monitoring of pressures is commonly used. Catheters can easily insert through the femoral vessels to measure arterial blood pressure or central venous pressure (2). The so-called Millar catheter is available in different versions and is being used for different applications. For experimental research different sizes allow its use in various animal species, from mice (3) to larger animals (4). Various types support measurement of physiological pressures, such as airway or cardiovascular pressures, such as left ventricular pressure (5, 6) or even electrophysiological applications. A Millar catheter also provides the possibility of continuous measurements of various cardiovascular parameters using pressure-volume loops (7). These are generated by plotting pressure and volume signals against each other in real time. A complete cardiac cycle is represented by one loop. Analyzing this loop, many parameters can be calculated in detail, e.g., Cardiac Output using stroke volume and heart rate.

Despite technical advance in recent years, functional imaging of the cardiovascular system in laboratory pigs, especially in neonatal piglets is still challenging. Magnetic resonance imaging (MRI) and computed tomography (CT) are often not readily available in every experimental setting. Furthermore, transport of anesthetized, catheterized and monitored animals to imaging facilities may affect study conditions and may influence results due to changes in body temperature or changes in ventilator or circulatory parameters. Therefore, using transthoracic echocardiography (TTE) may be a suitable option for point of care diagnostics.

TTE is used to assess hemodynamic status in critically ill patients with sepsis (8) or shock (9) on the ICU (10–12). Furthermore, echocardiography has been shown to have impact on patient management in intensive care units, specifically with regard to fluid administration and drug therapy (13–15). This has been effectively demonstrated in pediatric patients and neonates (16, 17). In addition, TTE allows to ensure the correct position of the used Millar catheters (5, 6, 18, 19). Therefore, transthoracic echocardiography may be extremely useful to for hemodynamic assessment in studies with animals, especially in small newborn animals (6). However, while the pig is a useful model, there are limitations in the use of TTE:

In contrast to humans, the swine has some important anatomic differences that affect acquisition of standard imaging planes and correct measurements. Its thorax configuration is rather “oval” in the anterior-posterior direction and the long axis of the heart is aligned perpendicularly to the long axis of the body (20). Due to small intercostal spaces and overlapping lung areas the acoustic window for TTE is small. As a result, some views routinely used in humans may be difficult or impossible to obtain. However, Fugelseth et al. and Odland et al. showed that TTE imaging of the laboratory piglet is possible (6, 21) using different positions. However, this is not always possible regarding to the study protocol. Therefore, the objective of our study was to investigate the feasibility of TTE in the very small newborn piglet in supine position only.

## Methods

### Animals/Procedures

All procedures conformed to the Society of Laboratory Animal Science (GV-SOLAS) as well as the National Animal Welfare Law. Following approval by the responsible government authority (Regierungspraesidium Tuebingen, Permit No. 1262) the procedures were performed according to the guidelines of the Federation of European Laboratory Animal Science Association (FELASA).

In the present study we performed transthoracic echocardiography in newborn piglets before, during and after asphyxia and hemorrhage. A detailed description on instrumentation/procedures is available in the original publication (2). Briefly, in this, forty-four anesthetized and intubated newborn piglets [crossing of pietrain and landrace; age 32 h (12–44 h), weight 1,220 g (1,060–1,495g,), median (IQR)] were exposed to standardized hypoxia and blood loss until asystole occurred. Blood loss was approximately one third of total estimated blood volume on average. The piglets were randomized into two groups to study different options of volume expansion and resuscitated according to ILCOR 2015 guidelines [including respiratory support, chest compressions (CC) and epinephrine use]. Return of Spontaneous Circulation (ROSC) was followed by a 4-h monitoring period. Four hours after ROSC piglets were euthanized using an intravenous overdose of potassium chloride during ongoing anesthesia with subsequent autopsy.

### Transthoracic Echocardiography

Echocardiography was performed at Baseline, 2 h and 4 h after ROSC by the same examiner experienced in human neonatal TTE in order to minimize potential bias. A point of care ultrasound device (M-Turbo^©^, FujiFilm SonoSite BV, Amsterdam, Netherlands) with an ultrasound transducer (P10x/8–4 MHz Sector Transducer, FujiFilm SonoSite BV, Amsterdam, Netherlands) was used. Animals were studied in supine position as mandated by the protocol of the original study. Before applying ultrasound gel, the skin was shaved. Additional procedures were not performed during TTE.

## Results

### 2D Imaging

**Figure 1:**
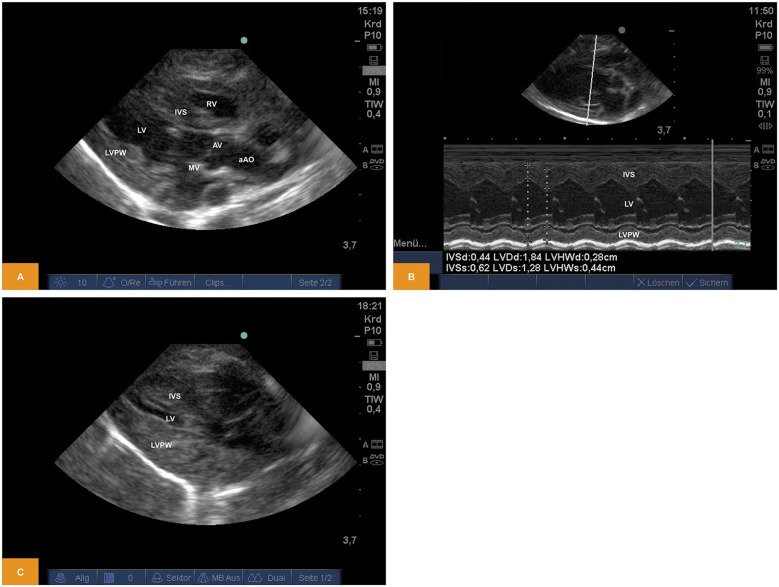
**(A)** Two-dimensional image of the left ventricular structures from PLAX (age: 70 h, weight: 1.45 kg). **(B)** M-Mode of the left ventricle obtained from PLAX (age: 70 h, weight: 1.45 kg). **(C)** Two-dimensional image of the left ventricular structures from PLAX after blood loss of approximately one third of blood volume (age: 12 h, weight: 1.40 kg). aAo, ascending aorta; AV, aortic valve; IVS, interventricular septum; LV, left ventricle; LVPW, left ventricular posterior wall; MV, mitral valve; PLAX, parasternal long axis; RV, right ventricle.

From a parasternal long axis view (PLAX), left-sided heart structures were visible in all imaging studies. Left atrium, the mitral valve, the muscular parts of the left ventricle (LV) and the left ventricular outflow tract (LVOT) with the aortic valve (AV) and parts of the ascending aorta (aAo) could be visualized. From the same view, functional imaging by M-mode was performed and measurements of interventricular septum (IVSd/s), left ventricular diameter (LVDd/s) and left ventricular posterior wall (LVPWd/s) were obtained. Left ventricular shortening fraction (SF) and ejection fraction (EF) were calculated (Table 1). Furthermore, there is the possibility to assess the volume status by visual observation of the filling of cardiac chambers (Figure 1).

**Table 1:** M-Mode Characteristics at Baseline: Values are presented as median (IQR); LVID, left ventricular internal diameter; IVS, interventricular septum; LVPW, left ventricular posterior wall; d, diastole; s, systole; EF, ejection fraction; SF, shortening fraction, *n* = 43; ^a^ =*n* = 40.

**LVIDd** **(mm)**	**LVIDs** **(mm)**	**IVSd** **(mm)**	**IVSs** **(mm)**	**LVPWd** **(mm)**	**LVPWs** **(mm)**	**EF** **(%)**	**SF** **(%)**
15.4 (13.9–17.4)	10.0 (8.6–11.2)	2.9 (2.6–3.4)	4.4 (3.9–4.9)	3.2 (2.8–3.6)	4.7 (4.3–5.3)	40^a^ (62–71)	34.5^a^ (30.4–37.4)

For illustration Videos 1, 2 are available as Supplementary Material.

### Doppler

**Figure 2:**
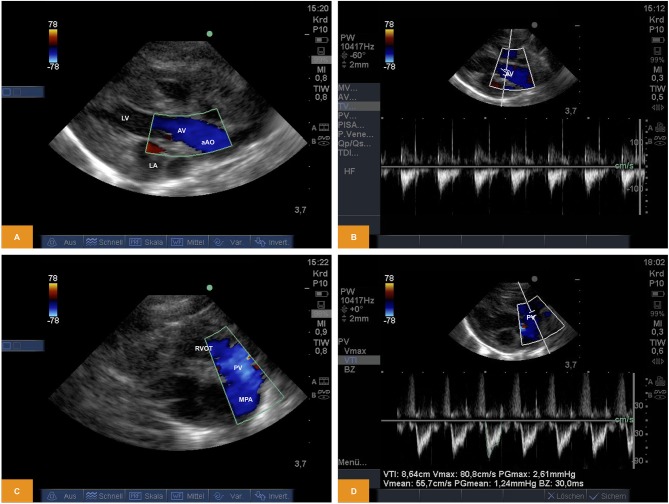
**(A,B)** Doppler study of flow across the aortic valve. The image was obtained from PLAX. Note the in favorable angle of insonation for measurement of pulsed wave Doppler (age: 70 h, weight: 1.45 kg). **(C,D)** Doppler study of flow across the pulmonary valve. The image was obtained from PSAX (age: 70 h, weight: 1.45 kg). aAo, ascending aorta; AV, aortic valve; LA, left atrium; LV, left ventricle; MPA, main pulmonary artery; PV, pulmonary valve; PLAX, parasternal long axis; PSAX, parasternal short axis; RV, right ventricle; RVOT, right ventricular outflow tract.

From PLAX left-sided heart structures were imaged as described above. Using Doppler sonography we were able to show/measure inflow into the left ventricle (LV) across the mitral valve (MV) and outflow across the aortic valve (AV). Using the parasternal short axis view (PSAX) the right ventricular outflow tract (RVOT) and flow across the pulmonary valve (PV) into the main pulmonary artery (MPA) becomes visible. Flow across the valves can be visualized by pulsed wave (PW) or continuous wave (CW) Doppler. Measurement of heart rate, peak and mean gradients or velocity time integral (VTI) are also possible, although the insonation angle is not favorable for Doppler studies across MV and AV and the measurements may significantly underestimate true values (Figure 2). For illustration Videos 3, 4 are available as Supplementary Material.

### Patent Ductus Arteriosus (PDA)

**Figure 3:**
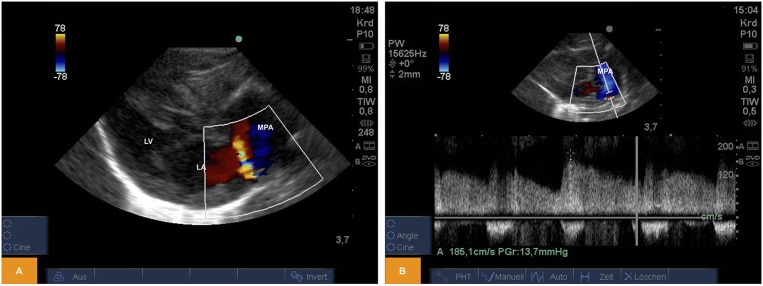
**(A)** Color Doppler image of left-to-right shunting across an arterial duct in a piglet from PSAX (age: 59 h, weight: 1.67 kg). **(B)** Pulsed wave Doppler signal of left-to-right shunting across an arterial duct from PSAX (age: 17 h, weight: 1.36 kg). LA, left atrium; LV, left ventricle; MPA, main pulmonary artery; PSAX, parasternal short axis.

From PSAX we could regularly detect shunting across an open arterial duct using color and pulsed wave Doppler in newborn piglets (Figure 3).

For illustration Video 5 is available as Supplementary Material.

### Congenital Anomalies

#### Insufficiency of Heart Valves

**Figure 4:**
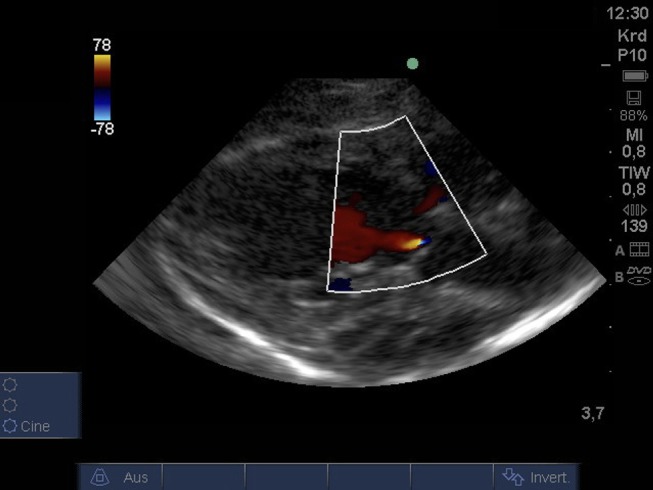
Color Doppler image of low-grade regurgitation across the aortic valve from PLAX (age: 36 h, weight: 1.48 kg). PLAX, parasternal long axis.

From PLAX detection of aortic insufficiency using color Doppler and 2D imaging was possible (Figure 4).

For illustration Video 6 is available as Supplementary Material.

#### Ventricular Septal Defect

**Figure 5:**
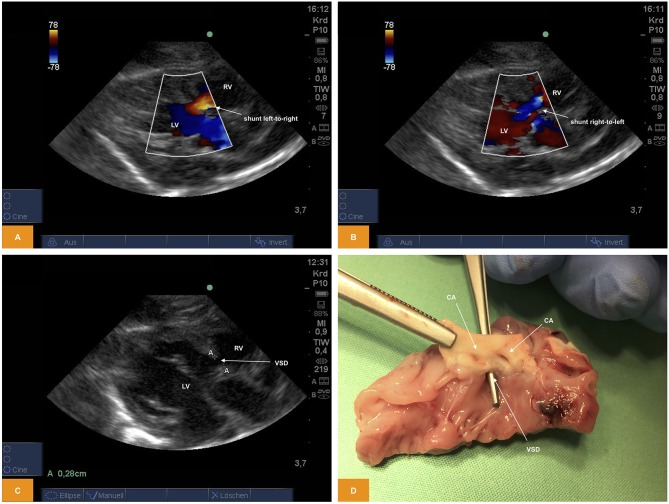
**(A,B)** Color Doppler image of shunting across a ventricular septal defect. The image was obtained from PLAX. Note the bidirectional direction of shunt with red representing left-to-right shunt and blue representing right-to-left shunt (age: 36 h, weight: 1.48 kg). **(C)** 2D image of a ventricular septal defect from PSAX (age: 36 h, weight: 1.48 kg). **(D)** Anatomic view of a ventricular septal defect (age: 36 h, weight: 1.48 kg). CA, coronary arteries; LV, left ventricle; PLAX, parasternal long axis; PSAX, parasternal short axis; RV, right ventricle; VSD, ventricular septal defect.

From PLAX we were able to prove presence of a ventricular septal defect with a size of ~2.8 mm using 2D imaging and a bidirectional shunt using color Doppler (Figure 5).

For illustration Video 7 is available as Supplementary Material.

### Various Other Aspects

**Figure 6:**
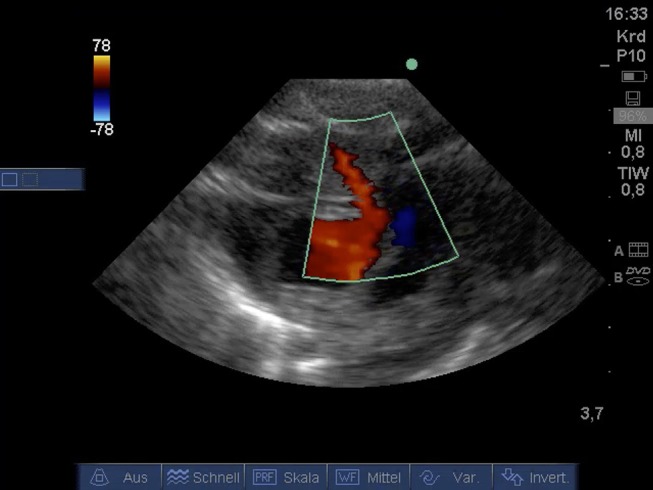
Color Doppler image of coronary artery from PSAX (age: 32 h, weight: 1.36 kg). CA, coronary artery; PSAX, parasternal short axis.

From PSAX detection of coronary arteries using color Doppler (Figure 6) was possible. A video for illustration is available as Supplemental Material Video 8.

Video 9 (available as Supplemental Material) shows detection of atrioventricular block using transthoracic echocardiography in piglet of 55 h of age and 1.69 kg of weight.

Furthermore, Video 10 (available as Supplemental Material) visualizes induction of cardiac arrest using an intravenous overdose of potassium chloride during ongoing anesthesia for euthanasia in piglet of 8 h of age and 0.93 kg of weight.

## Discussion

Kerut et al. showed in 2004 that transthoracic echocardiography is a useful modality for the imaging of cardiac structures in anesthetized older pigs (22). The feasibility of this method has been confirmed in a number of studies, and it may be considered a routine procedure at least in older/larger piglets. Lin et al. performed transthoracic echocardiography in Yorkshire piglets weighing 14.3 ± 4.1 kg in a study looking at the treatment of cardiac arrest and used short axis view and M-mode (23). Common crossbred piglets between 4 and 6 weeks of age and weighing 9.2 ±1.8 kg were used by Hayes et al. to visualize microbubbles (24). 3D Real-Time echocardiography was done by Herberg et al. in landrace piglets with a weight range about 3.6–8.0 kg (25). In 1999 the use of TTE was reported by Fugelseth et al. in newborn piglets with a mean weight about 1.6 kg (26). Odland et al. used tissue Doppler to study myocardial function during resuscitation and hypoxia in piglets with an average weight of 1.7 kg (21, 27, 28). In animals with approximately similar sized Børke et al. used strain Doppler echocardiography for the investigation of left ventricular function exposed to hypoxia (5). Therefore, our aim was to investigate feasibility of using TTE in newborn piglets with a slightly lower weight in supine position. We were able to visualize standard views such as PLAX or PSAX planes in all study piglets. Left-sided heart structures like left atrium, the mitral valve, the muscular parts of the left ventricle (LV) and the LVOT with the aortic valve (AV) and parts of the ascending aorta (aAo) could be visualized. From the same view, we were able to perform functional imaging by M-mode of the LV (Figures 1, 2). Left ventricular diameters could be measured to calculate shortening fraction. This may be useful for monitoring cardiac function in neonatal models of sepsis or cardiomyopathy or other entities affecting cardiac function. From PSAX the aortic valve could be imaged. In both views, there is possibility to determine the diameter of the aortic valve. This can be used for calculation of cardiac output as shown by Fugelseth et al., Fortin-Pellerin et al., and Solevåg et al. (6, 19, 29). Tilting toward the right side of the thorax allowed the RVOT, the pulmonary valve (PV) and the main pulmonary artery (MPA) to be visualized. The ductus arteriosus could be visualized using the color Doppler (Figure 3), which was also shown by Smith et al. (30). In their study, transthoracic echocardiography was performed in newborn piglets with weight similar to our animals, albeit TTE was only used to detect the ductus arteriosus. TTE was also used by Alvarez et al. (18) and Børke et al. in piglets with 1.6–2.7 kg in a strain Doppler echocardiography study (5), as well as Fugelseth et al. (6, 26) and Odland et al. (27) in animals of approximately the same weight to visualize or exclude an existing ductus arteriosus. In addition to the standard parameters mentioned above, TTE may be capable to detect congenital heart disease, including valve insufficiency or stenosis (Figure 4) or ventricular septal defect (Figure 5), as reported in somewhat larger animals by Børke et al. (5), and Fugelseth et al. (6, 26). The latter may be important to exclude animals with congenital heart disease from experimental studies to avoid bias when used for assessing respiratory or hemodynamic outcomes. Thus, screening for congenital heart disease with TTE may be important to limit variability of results in experimental studies. In summary, the standard views such as PLAX or PSAX planes can be visualized in very small piglets with a median weight of 1.2 kg. Induction of apnea was not necessary to achieve adequate image quality. Other TTE planes like four-chamber view, or subcostal and apical five-chamber planes can be visualized as well, but may need more experience, as shown by Solevåg et al. in piglets weighing ~2 kg (29) and shown by Fortin-Pellerin et al. (19) or Fugelseth et al (26). Other studies have shown that TTE can also be done in the right lateral, supine position as well as in the left lateral, half supine position (6, 26). Unfortunately, we were unable to assess positioning to access standard planes, because the protocol of the main study allowed supine position only (2). An additional limitation was that TTE was only performed on one piglet race using only one ultrasound probe. Furthermore, our animals were studied under general anesthesia, which may influence some measurement of cardiac function.

In summary, cardiac imaging by TTE in the laboratory piglet is a feasible method also in very small neonatal piglets in supine position. In our study ventricular structures could be visualized in parasternal long axis and parasternal short axis planes and functional parameters based on M-mode images could be measured.

## Data Availability

The datasets generated for this study are available on request to the corresponding author.

## Ethics Statement

This study was carried out in accordance with the recommendations of the Society of Laboratory Animal Science (GV-SOLAS) as well as the National Animal Welfare Law. The protocol was approved by the responsible government authority (Regierungspraesidium Tuebingen, Permit No. 1262) and the procedures were performed according to the guidelines of the Federation of European Laboratory Animal Science Association (FELASA).

## Author Contributions

SS, MK, and MM conceived and designed the study and also performed the animal experiments. SS and MK interpreted the results obtained and contributed equally to this research work. SS and MM drafted the manuscript. MM and HH revised the manuscript. All authors read and approved the final version of the manuscript and agreed to be accountable for all aspects of the work.

### Conflict of Interest Statement

The authors declare that the research was conducted in the absence of any commercial or financial relationships that could be construed as a potential conflict of interest.

## Supplementary Material

Video 1 Two-dimensional study of the left ventricular structures. Obtained from PLAX (age: 70 h, weight: 1.45 kg).

Video 2 Two-dimensional study of the left ventricular structures after blood loss of approximately one third of blood volume. Obtained from PLAX (age: 12 h, weight: 1.40 kg).

Video 3 Color Doppler study of flow across the aortic valve. Obtained from PLAX (age: 70 h, weight: 1.45 kg).

Video 4 Color Doppler study of flow across the pulmonary valve. Obtained from PSAX (age: 70 h, weight: 1.45 kg).

Video 5 Color Doppler study of a patent ductus arteriosus. Obtained from PSAX (age: 12 h, weight: 1.16 kg).

Video 6 Color Doppler study of an aortic insufficiency. Obtained from PLAX (age: 32 h, weight: 1.36 kg).

Video 7 Color Doppler study of shunting across a ventricular septal defect. Obtained from PLAX. Note the bidirectional direction of shunt with red representing left-to-right shunt and blue representing right-to-left shunt (age: 36 h, weight: 1.48 kg).

Video 8 Color Doppler study of a coronary artery. Obtained from PSAX (age: 32 h, weight: 1.36 kg).

Video 9 Two-dimensional study detecting atrioventricular block. Obtained from PLAX (age: 55 h, weight: 1.69 kg).

Video 10 Two-dimensional study during induction of cardiac arrest using an intravenous overdose of potassium chloride during ongoing anesthesia for euthanasia. Obtained from PLAX (age: 8 h, weight: 0.93 kg).
